# Heartworms in *Halichoerus grypus*: first records of *Acanthocheilonema spirocauda* (Onchocercidae; Filarioidea) in 2 grey seals from the North Sea

**DOI:** 10.1017/S0031182023000501

**Published:** 2023-08

**Authors:** Kristina Lehnert, Insa Herzog, Joy Ometere Boyi, Stephanie Gross, Peter Wohlsein, Christa Ewers, Ellen Prenger-Berninghoff, Ursula Siebert

**Affiliations:** 1 Institute for Terrestrial and Aquatic Wildlife Research, University of Veterinary Medicine Hannover; 2 Department of Pathology, University of Veterinary Medicine, Hannover,; 3 Institute of Hygiene and Infectious Diseases of Animals, Justus-Liebig-University Giessen, 35392 Giessen, Germany

**Keywords:** Harbour seal (*Phoca vitulina*), health monitoring, insect vector, molecular parasitology, seal louse (*Echinophthirius horridus)*, stranding network

## Abstract

The assumed definitive host of the heartworm *Acanthocheilonema spirocauda* (Onchocerdidae; Filarioidea) is the harbour seal (*Phoca vitulina*). This filaroid nematode parasitizing in cardiac ventricles and blood vessel lumina of harbour seals (*P. vitulina*) has a low prevalence and seldom causes severe health impacts. The seal louse (*Echinophthirius horridus*) is the assumed intermediate host for transmission of *A. spirocauda* filariae between seals, comprising a unique parasite assembly conveyed from the terrestrial ancestors of pinnipeds. Although grey seals (*Halichoerus grypus*) are infected by seal lice, heartworm infection was not verified. Analysing a longterm dataset compiled over decades (1996–2021) of health monitoring seals along the German coasts comprising post mortem investigations and archived parasites, 2 cases of *A. spirocauda* infected male grey seals were detected. Tentative morphological identification was confirmed with molecular tools by sequencing a section of mtDNA COI and comparing nucleotide data with available heartworm sequence. This is the first record of heartworm individuals collected from the heart of grey seals at necropsy. It remains puzzling why heartworm infection occur much less frequently in grey than in harbour seals, although both species use the same habitat, share mixed haul-outs and consume similar prey species. If transmission occurs directly *via* seal louse vectors on haul-outs, increasing seal populations in the North- and Baltic Sea could have density dependent effects on prevalence of heartworm and seal louse infections. It remains to be shown how species-specificity of filarial nematodes as well as immune system traits of grey seals influence infection patterns of *A. spirocauda*.

## Graphical abstract

**Figure:**
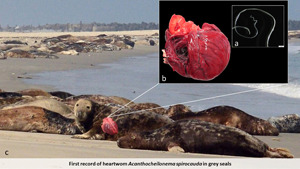

## Introduction

Grey seals (*Halichoerus grypus*) and harbour seals (*Phoca vitulina*) are the 2 resident seal species in the German North- and Baltic Sea with numbers increasing over the last decades (Reijnders *et al*., 2005; Galatius *et al*., 2020). In the last century grey and harbour seal populations had been depleted by hunting, habitat loss and contaminant exposure (Wolff, 2000; Lotze, 2007; Silva *et al*., 2021) in both the North- and Baltic Sea. Additionally, North Sea harbour seals were reduced by phocine distemper epidemics (1988 and 2002) (Härkönen *et al*., 2006) and influenza A virus, serotype H7N10 (2014) (Bodewes *et al*., 2015). Seals are infected by a multitude of parasites consisting mostly of trophically transmitted endoparasitic helminths with multi-stage life cycles and varying prevalences, intensities and pathological impacts. The heartworm *Acanthocheilonema* (*A*.) *spirocauda* is a filarial nematode (Onchocercidae; Filarioidea) that was first described from the heart of harbour seals (Leidy, 1858; Anderson, 1959) and later from the heart and surrounding blood vessels of multiple seal species including ringed (*Pusa hispida*), harp (*Phoca groenlandica*) and hooded seals (*Cristophora cristata*) (Measures *et al*., 1997) from Canada and North America (Dunn 1976). *A. spirocauda* is common in harbour seals along the coasts of The Netherlands (Van Den Broek and Wensvoort, 1959), Germany (Claussen *et al*., 1991; Lehnert *et al*., 2015), Denmark and Sweden (Lunneryd, 1992); see also Leidenberger, S. *et al*. (2007) for a review. Recently, *A. spirocauda* was recorded from monk seal (*Monachus monachus*) in the Mediterranean (Papadopoulos *et al*., 2010). The seal louse *Echinophthirius* (*E.*) *horridus* (Anoplura; Insecta) has been hypothesized to be the intermediate host for heartworm filariae that are transmitted *via* the blood meal and undergo several moults in the insect vector (Geraci *et al*., 1981; Lehnert *et al*., 2015; Ebmer *et al*., 2022) before becoming infective and being directly transmitted to a potential new host *via* infected louse vectors – transferred e.g. during haul-out on sandbanks between seal individuals. *A. spirocauda* was reported from the right chamber and atrium in harbour seals from the German Wadden Sea at 12% prevalence (Lehnert *et al*., 2015), while 25% (Borgsteede *et al*., 1991) and 32% prevalence (Claussen *et al*., 1991) were found after the 1988 PDV epidemic. Around the same time, 11% *A. spirocauda* prevalence was reported from the Kattegat/Skagerrak and Baltic Sea region (Lunneryd, 1992). Heartworm usually does not cause severe health effects in harbour seals, although a perforated atrium and subsequent mortality was described in 1 case (Lehnert *et al*., 2015). So far it was assumed that grey seals do not get infected by *A. spirocauda* (Leidenberger *et al*., 2007) although 1 study suggested the potential presence of heartworm in grey seal hosts (Keroack *et al*., 2018). This study reports *A. spirocauda* for the first time in the heart of 2 grey seals found stranded on the German coastline and complemented morphological identification of the specimens with molecular techniques for unambiguous species identity.

## Materials and methods

Within a coordinated stranding network established in 1990, post-mortem investigations on marine mammals found on the coasts of the German Federal State of Schleswig-Holstein (S-H) were performed at the Institute for Terrestrial and Aquatic Wildlife Research (ITAW) (Siebert *et al*., 2007). Dead stranded animals were retrieved and terminally sick animals were mercy-killed by licensed seal hunters for animal welfare reasons (Siebert *et al*., 2007). Decomposition status (DCC), with DCC1 being very fresh animals, DCC2 fresh, DCC3 moderate decomposition, DCC 4 advanced decomposition and DCC 5 mummified animals (Ijsseldijk *et al*., 2019) and nutritional status were assessed during necropsy which was performed in accordance to an established protocol (Ijsseldijk *et al*., 2019). Age was determined using dental growth layers. Carcasses were screened for ecto- and endoparasites and histopathological and microbiological investigations were conducted (Siebert *et al*., 2001, 2017). Prevalence and level of parasitic infections were determined during necropsy semi-quantitatively as none, mild, moderate or severe and associated lesions were preserved for subsequent histology (Lehnert *et al*., 2007; Siebert *et al*., 2007). During post-mortem investigation, the heart of the animal was separated from the lung, weighed and opened starting with the atrium and continuing with the ventricle of the according side. Parasites were collected in water and cleaned from tissue before being preserved in 70% ethanol. Associated lesions were assessed macroscopically and archived in 10% buffered formalin for histology. Grey seal (n = 164) necropsy findings and archived tissue samples from 25 years of health monitoring seals on the coasts of Schleswig-Holstein were screened in the frame of a long-term study between 1996 and 2021. Two male grey seals exhibited unusual heart nematode infections. The carcass of the first case was frozen before necropsy, the carcass of the second case was necropsied freshly. Collected nematode specimens were identified with a stereomicroscope (Olympus SDX10 and SX61 with CD30, Olympus, Hamburg, Germany) based on their morphological characteristics (Leidenberger and Boström, 2008). One adult female and one damaged heartworm specimen from the 2 different hosts were measured using CellSensEntry V3.2 software (Olympus, Hamburg, Germany). Because some isolated nematodes did not have unambiguous characteristics for parasite differentiation, species identification was achieved using gene sequence data. Genomic DNA was isolated from 2 specimens from the 2 grey seal individuals using a QIAamp DNA Micro Kit (Qiagen, Hilden, Germany). DNA concentrations and purity were determined using a Nanodrop 2000c (Thermo Scientific) spectrophotometer. Approximately 500 bp of the mtDNA COI gene was amplified using oligonucleotide primers 5`-GGTCCTGGGAGTAGCTGAAC-3` (forward) and 5`-ATGATGGCCCCACACAGAAG −3′ (reverse) (Lehnert *et al*., 2015). Polymerase chain reactions were performed in 50 *μ*L volume containing 25 *μ*L MyTaq Red Mix, 2x (Bioline, Heidelberg, Germany), 1 *μ*L of each primer (20 *μ*M), 5 *μ*L DNA template and distilled H_2_O to fill the volume. Cycling conditions were initial denaturation at 95°C for 1 min, followed by 40 cycles of denaturation at 95°C for 15 s, annealing at 60°C for 15 s and extension at 72°C for 10 s. PCR products were visualized on a 2.0% agarose gel using SYBRSafe DNA gel stain (Invitrogen, Germany). PCR products were Sanger sequenced at Microsynth (Göttingen, Germany). The closest match to the sequence was determined using BLASTN on GenBank.

## Results

**Figure 1.**
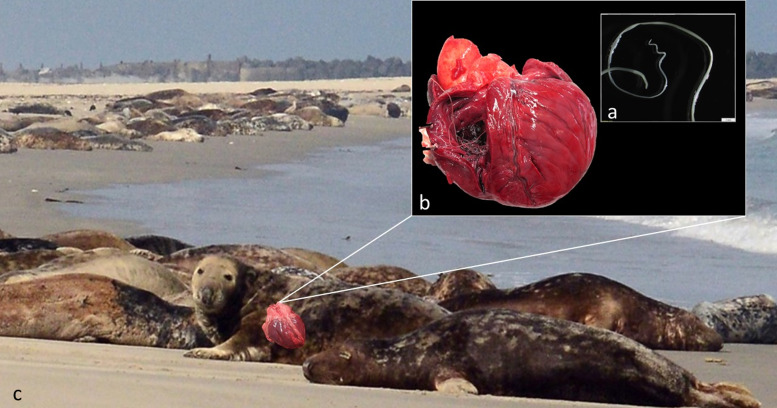
Heartworm *Acanthocheilonema spirocauda* with characteristic helical tail in adult male (a; inserted box) and in-situ right ventricle of seal heart (b; black box) found in grey seals (*Halichoerus grypus*) shown on haul-out on Helgoland (c).

Of 164 grey seals necropsied including the opening of the heart, 6 had samples of nematode infections in the heart. Four samples consisted of lungworm *Otostrongylus circumlitus* (Crenosomatidae; Metastrongyloidea), but heartworm samples originating from 2 grey seals found along the German North Sea coast were identified as *A. spirocauda*, a filarial nematode belonging to the Onchocercidae (Filarioidea) (Fig. 1).

The first heartworm sample originated from a 2-year-old male grey seal found in February 2018 on the island Heligoland. The animal was decomposition status grade 3 and in a good nutritional status with a body length of 147 cm. Due to predation the carcass displayed large wounds with missing skin, blubber and muscles and not all post mortem investigations could be performed. A moderate nematode infection of the left cardiac atrium and mild infections of the gastro-intestinal tract with anisakid nematodes and acanthocephalans were recorded at necropsy. A focal moderate granulomatous and eosinophilic mural enteritis with intralesional nematode larvae was observed histologically. Microbiological culture revealed no specific pathogens.

The second heartworm sample originated from a 17-year-old male grey seal found alive on a beach of the island of Sylt in January 2020. Due to its poor health condition, it was mercy-killed. The animal was in a moderate nutritional status with a total length of 194 cm and a body weight of 114 kg. At necropsy 1 day later (decomposition status 1) a single nematode was collected from the heart. Severe infections with gastric anisakid nematodes and *Corynosoma* spp. acanthocephalans in the small intestine as well as with respiratory mites *Halarachne halichoeri* were observed. In histology a moderate, granulomatous, mural gastritis with intralesional parasites as well as a marked eosinophilic and granulomatous enteritis accompanied by a similar lymphadenitis of the mesenteric lymph nodes were diagnosed. Additionally, pulmonary and gastric lymph nodes showed a mild follicular hyperplasia. A mild purulent valvular endocarditis was observed possibly indicating a septicaemic origin. Microbiological investigation revealed the presence of *Streptococcus equi* subspecies *zooepidemicus* in nearly every organ sample. Only single colonies were detected in the pulmonary lymph node, intestine and intestinal lymph node, low growth rates in spleen, kidney, stomach and lung and moderate growth rate in the central nervous system. Strong growth of *Clostridium perfringens* was demonstrated in the intestine.

In both samples from the heart the nematode individuals appeared slender and whitish with firm rounded shape of the cuticula. Posterior body ends of males with characteristic helical tail and spicules for unambiguous morphological identification were not observed. In 1 sample the length of an intact female specimen was 9.2 cm, with a width of 0.5 mm. Other specimens were incomplete and in some instances entangled. The second sample contained only 1 damaged specimen of undetermined sex with a length of at least 12.3 cm and a width of 0.9 mm. The 474 bp long mtDNA sequence derived from the nematodes after PCR was 99.79% identical with *A. spirocauda* published sequence of the COI gene for cytochrome oxidase subunit 1 when blasted in GenBank (accession no.: HF583266.1).

## Discussion

Nematodes collected from the heart of 2 male grey seals found along the German North Sea coast were molecularly identified as *A. spirocauda* and comprise the first record of this species in grey seals during a decades-long monitoring of seal health along the German North- and Baltic Sea coast. Heartworms are common in harbour seals and frequently infect individuals found stranded along the German North- and Baltic Sea coasts (Claussen *et al*., 1991; Lehnert *et al*., 2007, 2015) and adjacent waters (Lunneryd, 1992). However, they were so far not reported from grey seals which share their habitat, diet preferences and haul-outs with harbour seals (Brasseur, 2017; Damseaux *et al*., 2021; Boyi *et al*., 2022). The insect seal louse *Echinophthirius* (*E*.) *horridus* infects both harbour and grey seals and occur regularly on both species throughout the study area (Zimmermann and Nebel, 1975; Lehnert *et al*., 2015) and in adjacent waters (Thompson *et al*., 1998; Morick *et al*., 2009). Although *E. horridus* is assumed to be an intermediate host and vector of the heartworm filariae (Geraci *et al*., 1981; Leidenberger *et al*., 2007; Lehnert *et al*., 2015), it was believed that heartworms do not infect grey seals (Measures *et al*., 1997; Leidenberger *et al*., 2007). A recent study found circumstantial evidence for *A. spirocauda* infecting grey seals by using a qPCR assay for heartworm infections, detecting a positive signal from a damaged nematode sampled from a seal carcass, assumed to be a grey seal but due to decomposition it was not possible to specify the host (Keroack *et al*., 2018). However, this is the first study actually reporting heartworm individuals found in the heart of grey seals at necropsy.

Lungworms belonging to the Metastrongyloidea usually infect the respiratory tract (Measures, 2001) but are frequently found in the heart and surrounding blood vessels of seals at necropsy (Claussen *et al*., 1991) and can be mistaken for *A. spirocauda*, necessitating a thorough morphological or molecular identification. *Otostrongylus* (*O*.) (Crenosomatidae) and *Parafilaroides* (*P*.) *gymnurus* (Filaroididae) are found more seldom in grey seals compared to harbour seals, where prevalences of 70% are common (Lehnert *et al*., 2007). The morphology of *A. spirocauda* (Onchocercidae) and 2 occurring lung nematode species *O. circumlitus* and *P. gymnurus* in grey seals is distinct. *O. circumlitus* lungworms are thicker, with their cuticula more crumpled and beige in coloration, *P. gymnurus* are significantly smaller and more fragile than *A. spirocauda*. Especially adult male nematodes of the mentioned species display clear species-specific morphological traits in the posterior body end. Length and width measurements of an adult *A. spirocauda* specimen found in grey seals in this study corresponded with previous measurements of *A. spirocauda* found in harbour seals (Leidy, 1858) also from the North Sea (Wipper, 1974; Van der Kamp, 1987). However, it is challenging to differentiate damaged or larval nematode specimens. The emergence of molecular tools to differentiate between and speciate parasites without clear morphological traits enabled the identification of *A. spirocauda* with mitochondrial nucleotide data in this study. Based on the molecular data obtained, harbour and grey seals are infected by the same *Acanthocheilonema* species (*A. spirocauda*). Also, the lack of nucleotide difference between the grey and harbour seal heartworm shows that *A. spirocauda* of the same haplotype infects both species and the heartworm has not developed genetic adaptations to either species.

The low occurrence and intensity of *A. spirocauda* infections in grey seals in the study area over the last decades indicates a high species-specificity of heartworm as designated parasite of harbour seals. However, while seal populations have steadily increased over the last years (Olsen *et al*., 2018) and grey seals have recolonized the German North- and Baltic Sea after almost becoming extinct in the last century (Reijnders *et al*., 2005), inter-species contacts and density-dependent infection patterns may influence the prevalence and intensity of *A. spirocauda* in both host species (Reckendorf *et al*., 2019). In the investigated grey seals heartworm infections were mild and moderate and probably did not substantially contribute to the cause of death or disease. However, both individuals had gastro-intestinal parasite infections, in 1 grey seal severe gastro-intestinal helminth and respiratory mite infections which debilitated the animal and probably contributed to its generalized infection. The detection of *S. equi* subspecies zooepidemicus in various organ samples together with the diagnosis of an endocarditis indicate that septicaemia might have been the cause of severe disease in this grey seal. The single heartworm encountered could also have caused mechanical alteration of the endothelium of the valve and provoked acute endocarditis. Although heartworms have been described to cause obstructions (Dunn and Wolke, 1976; Stroud and Dailey, 1978) in harbour seals, mechanical alterations like a perforation of the right atrium severely infected by *A. spirocauda* are scarce (Lehnert *et al*., 2007).

The traumatic lesions in the first individual are assumedly caused by predation of another grey seal (Van Neer *et al*., 2020). Microbiological findings in the lung need to be judged with caution as the thorax was opened due to the predation wound and thoracic organs were probably contaminated from the outside. Inflammatory alterations in the intestine observed in histology were possibly caused by the parasitic infection. The parasites in the intestinal wall of both grey seals diagnosed in histology indicate the somatic migration of nematode larvae, however, it remains unclear if this may be heartworm, gastro-intestinal or other parasite species. Although heartworms seem to have mild pathogenicity in harbour seals – with mild infections and no significant impact on health apart from some cases (Dunn and Wolke, 1976; Stroud and Dailey, 1978; Conlogue *et al*., 1980; Lehnert *et al*., 2007) their pathogenicity in grey seals remains to be evaluated pending on more cases. On both grey seals no seal lice (*E. horridus*) were found, although they are suspected to be vectors of heartworm filariae (Geraci *et al*., 1981). However, ectoparasites can leave their host after death, or may get lost during stranding, drifting or transport of the carcass, therefore introducing a bias into ectoparasite prevalence. It remains striking that although harbour and grey seals are closely related, share the same ecosystem and many resources, grey seals are not similarly affected by certain infectious diseases, e. g. phocine distemper virus (PDV), lung nematode (Metastrongyloidea) (Osinga *et al*., 2012) and heartworm infections. Heterozygosity has been suggested to cause varying susceptibility to infectious disease (McCarthy *et al*., 2011) including lung nematodes (Rijks *et al*., 2008) within and among host species in natural harbour seal populations (Hoffman *et al*., 2014) when new approaches of uncovering heterozygosity fitness correlations for varying fitness within a population were investigated. Consequently heterozygosity could also be considered as possible factor explaining susceptibility to nematode infections in harbour and grey seals. Long term data sets and sample archives with preserved biological specimens and tissues collected over extended periods of time are especially valuable when investigating long-lived apex predators and effects of environmental change on their ecosystem (Reckendorf *et al*., 2019; Wood and Vanhove, 2022). Parasites can serve as important bio indicators for the ecology of their hosts, reflecting behaviour, diet and distribution (Marcogliese, 2005; Hudson *et al*., 2006), and, in the case of grey seals potentially their reestablishment and subsequent richer biodiversity in the North Sea ecosystem (Marcogliese, 2004). The intricate parasite-host relationships between heartworm and seals and the species-specificity of *A. spirocauda* as well as grey seal immune traits need to be investigated further.
